# Supplementary material for: MAHLER: Integrating Metadynamics and Inverse Folding to Predict Antibody-Antigen Kinetics
Source: bioRxiv. 2026 Jun 12:2026.06.10.731383. Preprint. [Version 1] doi: 10.64898/2026.06.10.731383 (PMC13277939; doi:10.64898/2026.06.10.731383)
Supplement: Supplementary file 1 [file NIHPP2026.06.10.731383v1-supplement-1.pdf]

bioRxiv preprint doi: https://doi.org/10.64898/2026.06.10.731383; this version posted June 12, 2026. The copyright holder for this preprint (which was not certified by peer review) is the author/funder, who has granted bioRxiv a license to display the preprint in perpetuity. It is made available under a CC-BY 4.0 International license.

Supplementary Information

A Supplementary Discussions

A.1 On selection of CVs and exploratory MetaD

As noted in the Methods section, we noted that collective variables (CVs) such as the Q-value or AgAb distance are often inappropriate for rigorous enhanced sampling. While the definition of an "ideal" CV depends on specific sampling or analysis goals, a fundamental requirement is the ability to resolve distinct metastable states[56].

By this metric, geometric descriptors like RMSD, antigen-antibody distance, or the fraction of native contacts are often suboptimal. These variables typically suffer from high and heterogeneous degeneracy; multiple distinct metastable states can map to the exact same scalar value (e.g., the same RMSD or distance). Consequently, these CVs frequently fail to capture the system's slowest modes, leading to hysteresis and poor convergence.

In this work, we employed the interface residue distance and interface Q-value strictly to drive the system toward dissociation, not to recover a converged free energy surface, or for kinetics calculation. These variables do not guarantee that the system visits all relevant intermediate states.

This limitation is evident in our 30 replicas of exploratory MetaD (Figure S1), where the bias potentials deposited to induce dissociation varied significantly in both height and shape across replicas. Such variance indicates insufficient sampling of orthogonal degrees of freedom. For reference, these simulations used a Gaussian bias with a height of 0.3 kcal/mol, widths of 0.2 Å (distance) and 0.02 (Q-value), and a bias factor of 15.

A.2 On the choice of key atoms

The feature selection algorithm is detailed in Ref. 23; here, we summarize its specific application to this system.

We define a set of key atoms comprising N_g atoms from the antigen and N_b atoms from the antibody, generating a total of $N_g N_b$ pairwise distances. To identify features that capture the conformational diversity predicted by AF-M, we rank these distances by their coefficient of variation (standard deviation divided by the mean) across the generated ensemble. The top s features (here, $s = 200$) are used to cluster the conformations. From this reduced pool, the AMINO algorithm identifies a sparse, non-redundant subset (here, 19 distances) to learn the latent space.

For the candidate pool, we selected the C_α atoms of all interface residues (as defined in Methods). We restricted the selection to surface atoms for two physical reasons. First, the antibody core is generally rigid, so biasing long-range distances involving core atoms risks forcing unphysical deformations rather than valid conformational changes. Second, distances involving atoms away from the interface are typically much longer than those between interface pairs. Geometrically, when the radial degree of freedom is fixed, a larger radius results in a larger spherical shell. This increased angular freedom introduces higher degeneracy, rendering long-range distances less informative for distinguishing specific metastable states.

bioRxiv preprint doi: https://doi.org/10.64898/2026.06.10.731383; this version posted June 12, 2026. The copyright holder for this preprint (which was not certified by peer review) is the author/funder, who has granted bioRxiv a license to display the preprint in perpetuity. It is made available under a CC-BY 4.0 International license.

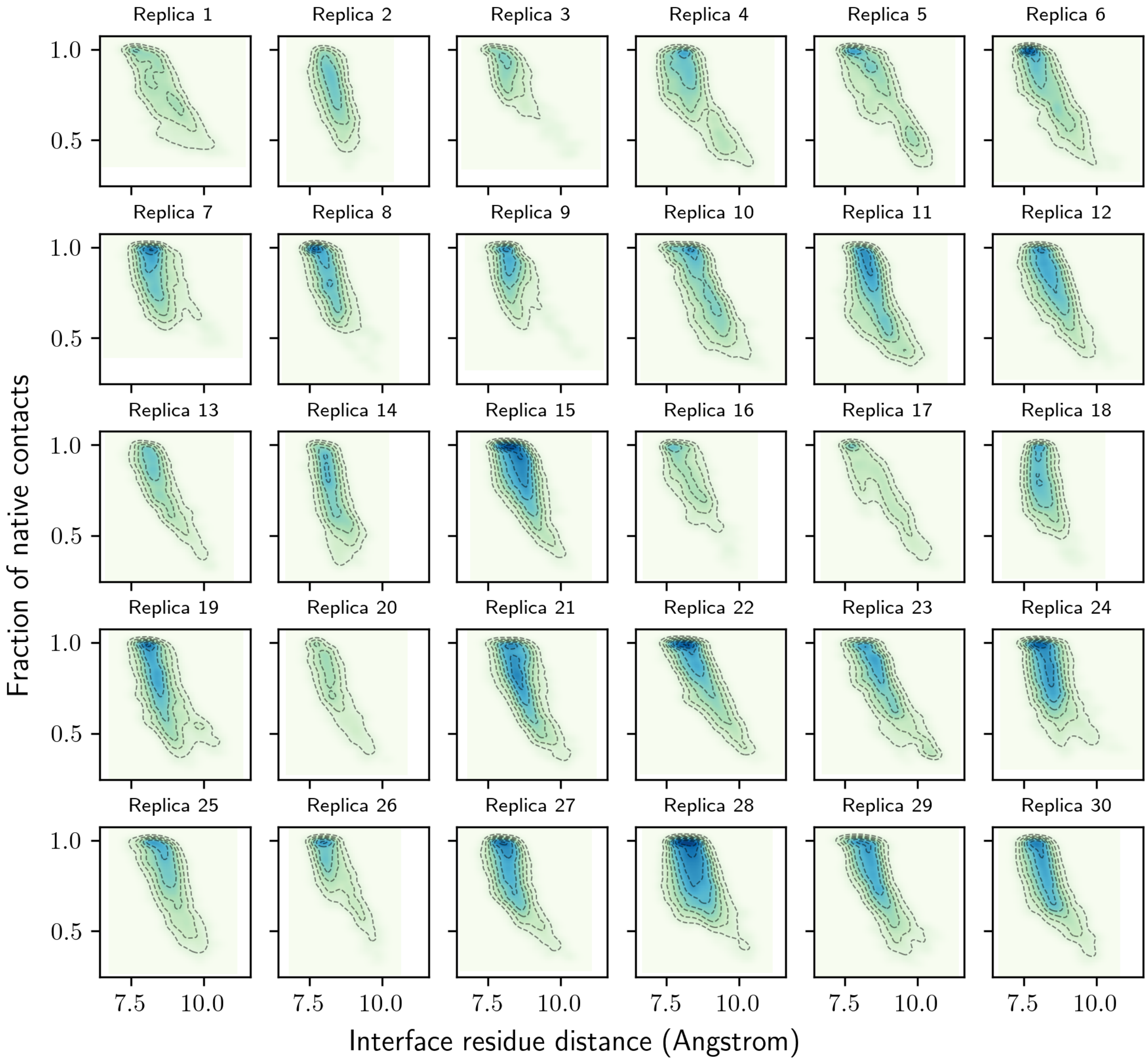

Figure S1. Hills deposited for the exploratory MetaD. 30 replicas of exploratory MetaD were run for 2CMR and were projected onto the latent space. Contour lines are spaced 1.0 kcal/mol away from each other. Color intensity corresponds to the cumulative density of deposited bias

B Supplementary Methods

B.1 Structures generated with AlphaFold-Multimer

We projected the structures generated by AF-M by several metrics. A few conclusions can be drawn from these results.

First, across the three accuracy predictors provided by AF-M, none of them is decisive in determining the correctness of the structure (Fig. S2), characterized by a high interface Q-value. Even ipTM scores are noisy in this sense. RMSD to experimental structures as a metric often ignores the existence of alternative binding epitopes.

Second, the dependency of structural quality, characterized by steric clashes, is complicated in terms of MSA depth (Fig. S3).

Different MSA depths give very different patterns. For example, full MSA tends to give higher quality

bioRxiv preprint doi: https://doi.org/10.64898/2026.06.10.731383; this version posted June 12, 2026. The copyright holder for this preprint (which was not certified by peer review) is the author/funder, who has granted bioRxiv a license to display the preprint in perpetuity. It is made available under a CC-BY 4.0 International license.

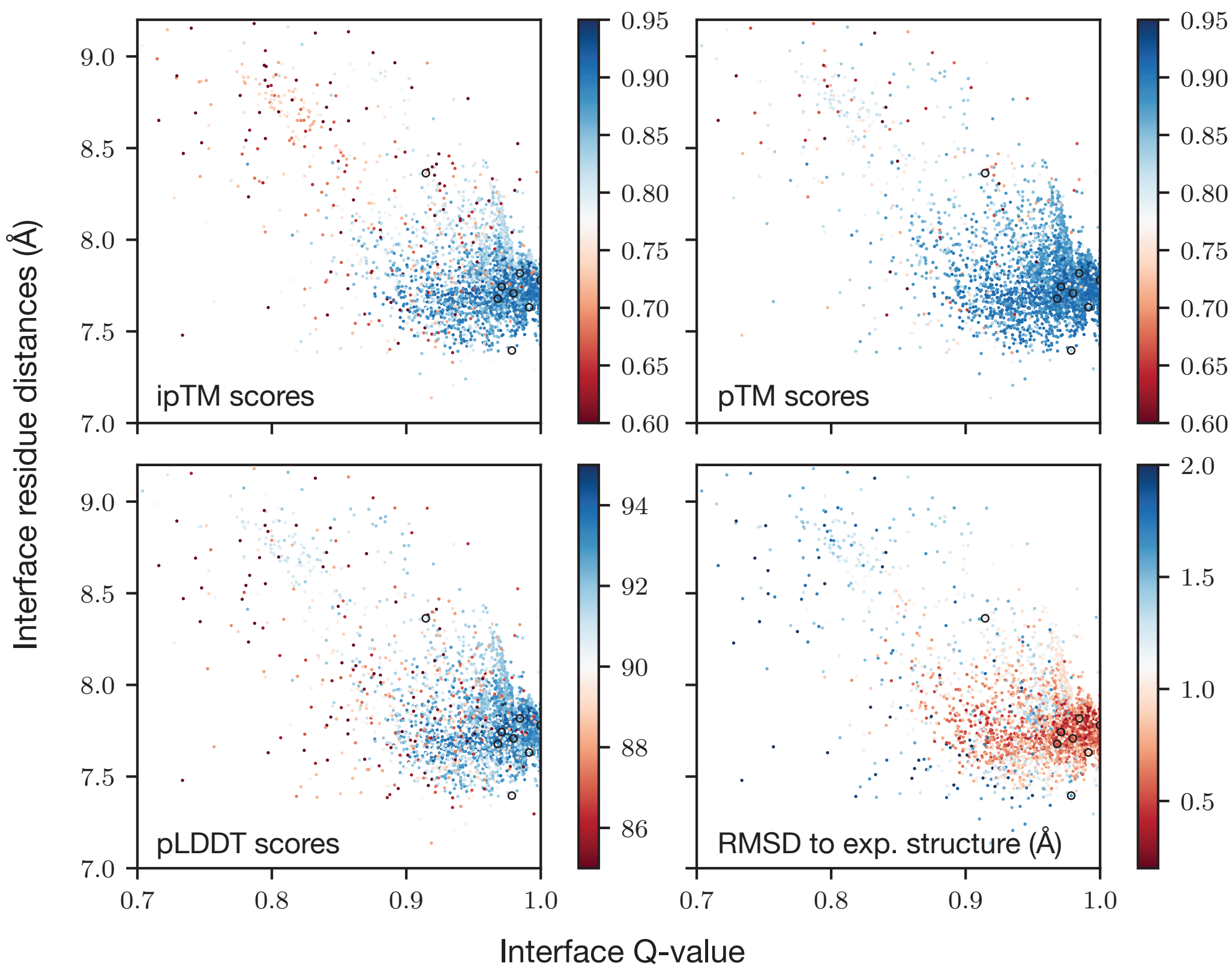

Figure S2. **Qualities of AF-M generated 2CMR structures.** AF-M can generate slightly dissociated structures, bypassing the need for additional sampling. All structures that passed the steric clash filter were projected by the interface Q-value and interface residue distances. Selected representative structures are shown in black circles. Three AF-M predicted metrics (ipTM, pTM, and pLDDT) were used to color the points. Black circles show the selected seed structures for MD.

structures in all three metrics. Shallower MSA usually gives a longer tail in these figures, but nearly none of them pass the steric clash filter. It shows that there is probably not a single "best" MSA depth to gain diversity.

Despite this longer tail, it doesn't mean shallower MSA necessarily is better at discovering alternative modes, as demonstrated in the case of ADK in Ref. 23. For example, two "islands" of structures that are around 8 Å and 11–16 Å away from experimental structures appeared only in full MSA predictions. However, none of them passed the steric clash filter as well, indicating a clashing binding epitope.

bioRxiv preprint doi: https://doi.org/10.64898/2026.06.10.731383; this version posted June 12, 2026. The copyright holder for this preprint (which was not certified by peer review) is the author/funder, who has granted bioRxiv a license to display the preprint in perpetuity. It is made available under a CC-BY 4.0 International license.

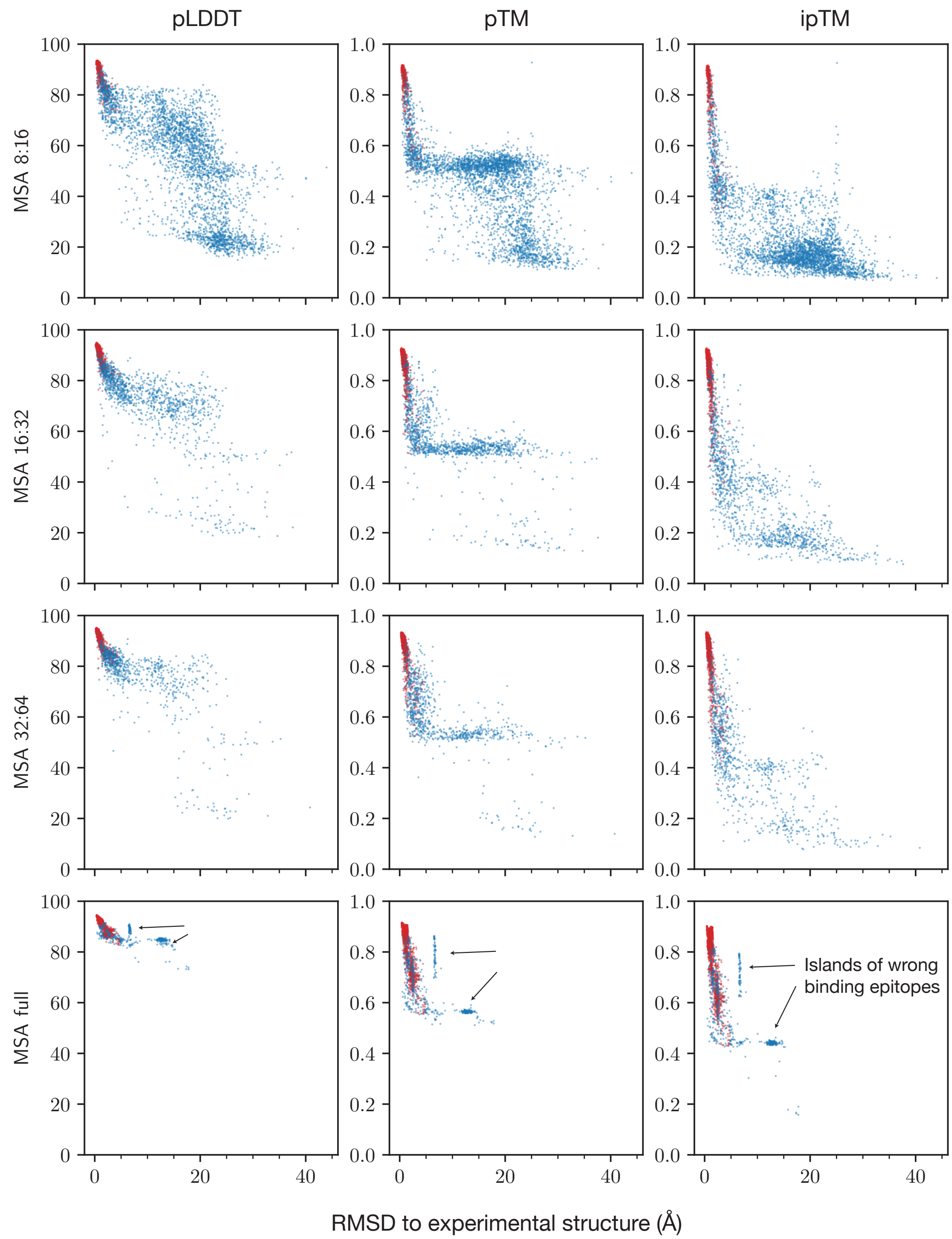

Figure S3. **Effect of MSA depth and steric clash filter.** This figure provides a profile of the generated structures. The red points are those passing the steric clash filter at 1 Å.

B.2 Extrapolation of SPIB latent space

Here in Figure S4, we report the projection of the 30 exploratory MetaD trajectories from 2CMR in the latent space, colored by fraction of native interface contacts. Here we juxtaposed the same projection colored by interface residue distances (left) and the and fraction of native contacts (right).

Our results show that both in terms of interface residue distances (d^{IR}) and Q-value, our latent space projects well, as a supplement to Fig. 1c.

bioRxiv preprint doi: https://doi.org/10.64898/2026.06.10.731383; this version posted June 12, 2026. The copyright holder for this preprint (which was not certified by peer review) is the author/funder, who has granted bioRxiv a license to display the preprint in perpetuity. It is made available under a CC-BY 4.0 International license.

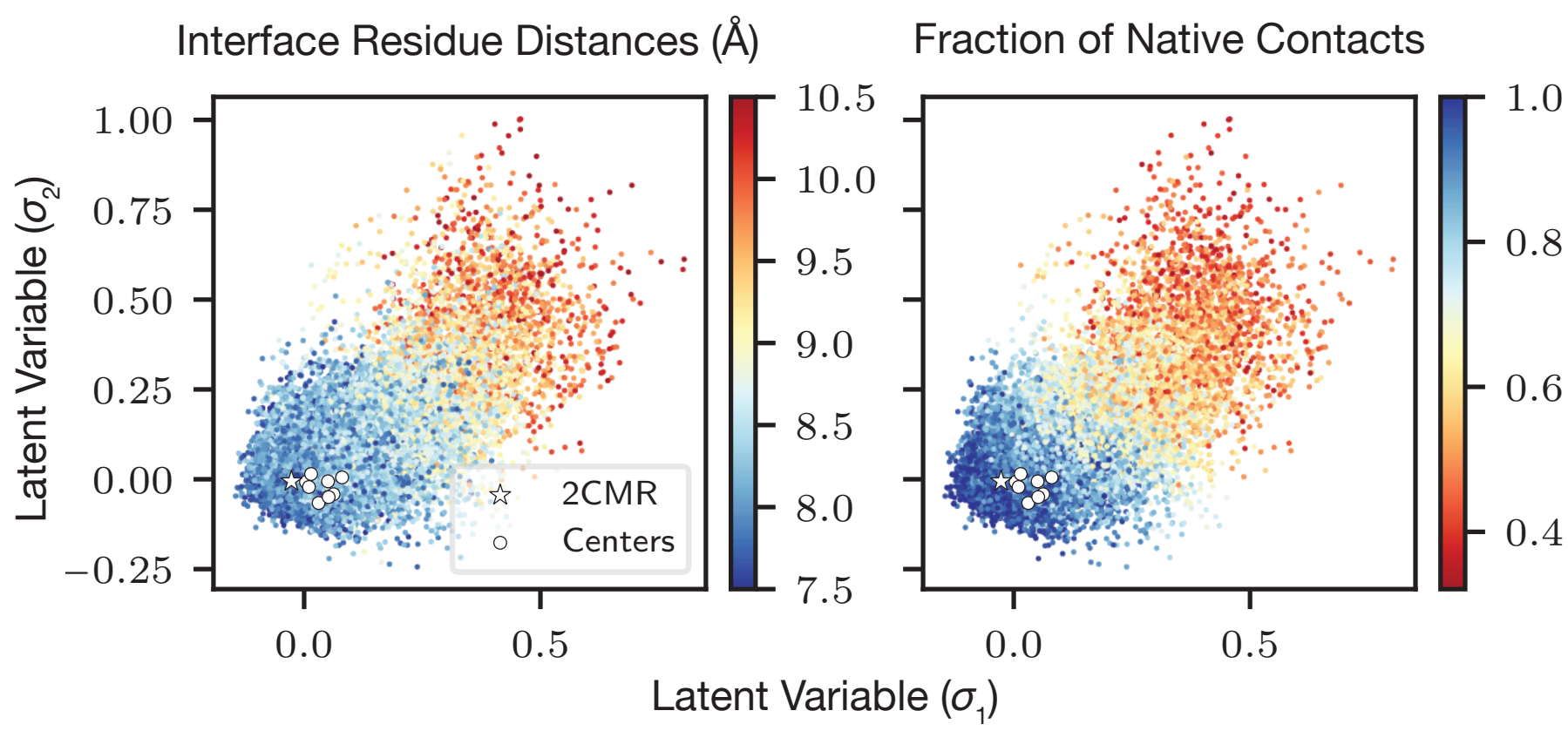

Figure S4. **Projection of exploratory 2CMR MetaD trajectories on SPIB latent space.**

B.3 Mean score calculation

Figure 3b showed the mean score from three unbiased MD runs. Trajectories were saved every 0.1 ns and scored with ProteinMPNN. The relative score $\{s_i\}$ from ProteinMPNN is a log-likelihood.

$$s_i = -\ln \frac{P^{\text{mut}}(x)}{P^{\text{WT}}(x)} \tag{7}$$

When setting $V = 0$ and that $e^{\beta V} = 1$ in Equation (6), a meaningful mean value can be calculated as

$$\langle s \rangle = -\ln \left(\frac{1}{N} \sum_i e^{-s_i} \right). \tag{8}$$

bioRxiv preprint doi: https://doi.org/10.64898/2026.06.10.731383; this version posted June 12, 2026. The copyright holder for this preprint (which was not certified by peer review) is the author/funder, who has granted bioRxiv a license to display the preprint in perpetuity. It is made available under a CC-BY 4.0 International license.

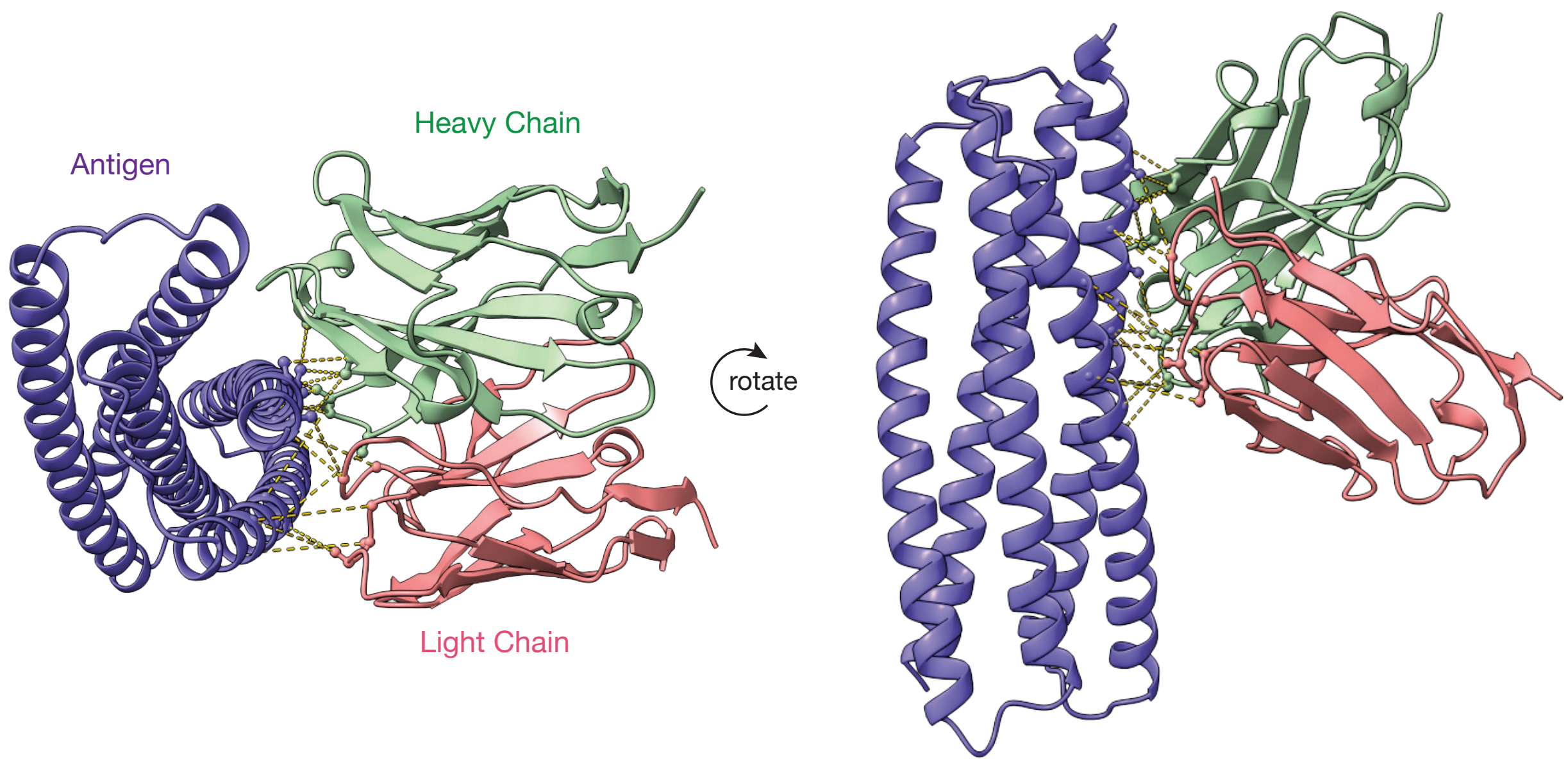


```
           1   5   10   15   20   25   30   35   40   45   50
           MQLLSGIVQQQNNLLRAIEAQQHLLQLTVWGIKQLQARILAGGSGGHTTW
           MEWDREINNYTSLIHSLIEESQNQQEKNEQELLEGSSGGQLLSGIVQQQN
Antigen    NLLRAIEAQQHLLQLTVWGIKQLQARILAGGSGGHTTWMEWDREINNYTS
           LIHSLIEESQNQQEKNEQELLEGSSGGQLLSGIVQQQNNLLRAIEAQQHL
           LQLTVWGIKQLQARILAGGHHHHHHG
           DIQMTQSPSTLSASIGDRVTITCRASEGIYHWLAWYQQKPGKAPKLLIYK
Light Chain ASSLASGAPSRFSGSGSGTDFTLTISSLQPDDFATYYCQQYSNYPLTFGG
           GTKLEIK
           QVQLVQSGAEVRKPGASVKVSCKASGDTFSSYAISWVRQAPGQGLEWMGG
Heavy Chain IIPIFGTANYAQAFQGRVTITANESTSTAYMELSSLRSEDTAIYYCARDN
           PTLLGSDYWGAGTLVTVSS
```

A (Bold): Involved in features
A (Underline): Interface residues

Figure S5. Sequence and interface of the 2CMR system. Upper: visualization of the 19 distances used as features. Lower: sequence for the system and key residues involved.
